# Commonly used indices disagree about the effect of moisture on heat stress

**DOI:** 10.1038/s41612-023-00408-0

**Published:** 2023-07-05

**Authors:** Charles H. Simpson, Oscar Brousse, Kristie L. Ebi, Clare Heaviside

**Affiliations:** 1 Institute of Environmental Design and Engineering, Bartlett School of Environment Energy and Resources, University College London, 14 Upper Woburn Place, London, UK; 2 Center for Health and the Global Environment, University of Washington, Seattle, WA, USA

## Abstract

Irrigation and urban greening can mitigate extreme temperatures and reduce adverse health impacts from heat. However, some recent studies suggest these interventions could actually exacerbate heat stress by increasing humidity. These studies use different heat stress indices (HSIs), hindering intercomparisons of the relative roles of temperature and humidity. Our method uses calculus of variations to compare the sensitivity of HSIs to temperature and humidity, independent of HSI units. We explain the properties of different HSIs and identify conditions under which they disagree. We highlight recent studies where the use of different HSIs could have led to opposite conclusions. Our findings have significant implications for the evaluation of irrigation and urban greening as adaptive responses to overheating and climate adaptation measures in general. We urge researchers to be critical in their choice of HSIs, especially in relation to health outcomes; our method provides a useful tool for making informed comparisons.

## Introduction

In hot atmospheric conditions and under exertion, the human body must dissipate heat to maintain its internal temperature, which is mainly achieved by perspiration. High atmospheric humidity can limit the rate of evaporation of sweat and, therefore, the amount of heat dissipated, so increasing heat stress on the body^1^. The concept of moist heat stress is increasingly used in research into the health effects of urban heat and climate change^2^.

Atmospheric conditions that lead to heat stress are often assessed using heat stress indices (HSIs). Most HSIs combine temperature and humidity in some way (e.g., the heat index (HI) used by the National Oceanic and Atmospheric Administration (NOAA)), and some incorporate thermal radiation and wind speed (e.g. universal thermal climate index (UTCI))^3^. Choosing the right index is crucial, as it sets the relative importance of temperature, humidity, and any further quantities in determining heat stress and, therefore, health outcomes. There is not usually a one-to-one mapping between different HSIs, and they do not operate on a common scale (even if both use °C); this makes it impossible to make direct comparisons between the values of different HSIs.

Many studies have noted that drier conditions lead to higher air temperature, often exacerbating heatwave conditions^4–6^; others have modelled or observed the tendency of irrigated agricultural land to reduce air temperature^7–9^. Thiery et al.^10^ found that irrigation expansion has substantially offset the effect of global warming on extreme temperatures in some regions. These relationships point to the conclusion that a drier land surface leads to worsened heat stress. Conversely, some recent studies have argued that droughts generally decrease heat stress because of the effect of reduced humidity^11^, while irrigation increases heat stress because of the effect of increased humidity^12–15^. This discrepancy has potentially major implications, as some climate adaptation actions intended to reduce heat stress, such as urban greening, rely on the conversion of sensible heat to latent heat through evapotranspiration of water. However, we will show that the arguments in these studies are highly subject to the choice of HSI used so further examination of the correct use of HSIs is imperative.

In this study, we focus on a selection of HSIs commonly used in heat-health studies: WBT, “indoor” wet-bulb globe temperature (WBGT), simplified WBGT (sWBGT), apparent temperature^16^, NOAA’s HI, humidex and UTCI. The effects of radiation are often neglected in heat-health studies^17^, which may be because radiation data are less widely available, seen as requiring more assumptions, or are seen as less reliable. In this paper, we, therefore, only focus on the relative effects of temperature and humidity.

While previous studies have identified that the emphasis of humidity in heat stress depends on choice HSI^2,18^, in this study, we put these comparisons on a strong quantitative footing. We demonstrate a method that uses calculus of variations to estimate the relative marginal sensitivity of different HSIs to air temperature and humidity on a common scale and therefore identifies the atmospheric conditions under which HSIs will disagree with each other. This addresses a major knowledge gap in climate-health studies. We identify examples where the choice of HSI reverses the conclusions on the effect of joint changes in temperature and humidity (caused in these examples by changes in soil moisture) on heat stress.

## Results and Discussion

### When do commonly used indices agree?

The range of temperature and humidity for which different HSIs are inconsistent can be identified through the calculus of variations without any need to perform atmospheric or land surface modelling. We use the example of UTCI and WBT to explain our method of comparison between HSIs but expand to the comparison of more HSIs in a later section.

**Fig. 1:**
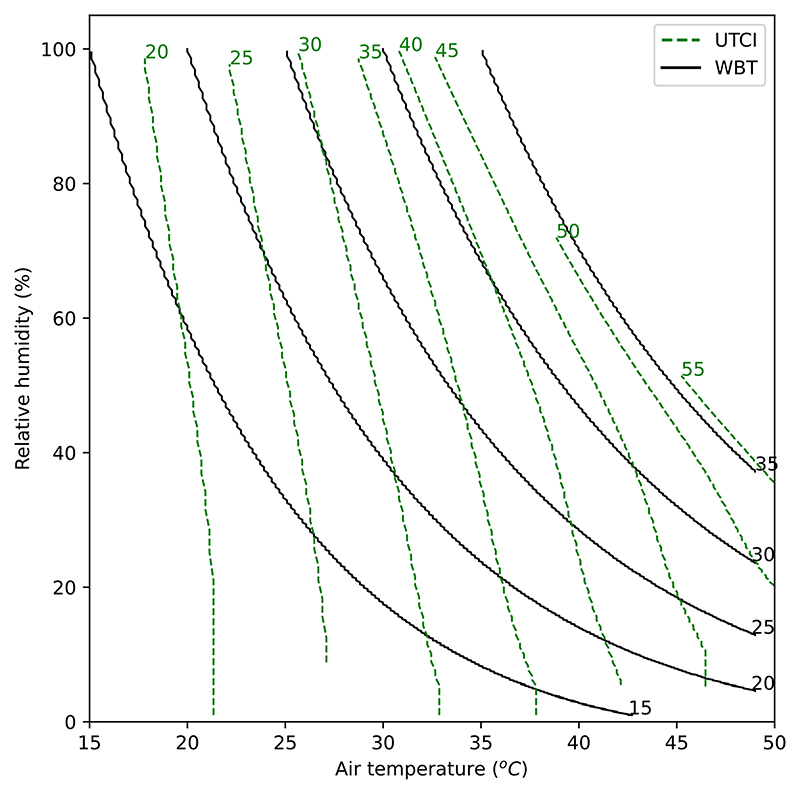
Isopleths of UTCI and WBT in temperature relative-humidity space. Dashed green lines show UTCI, solid black lines show WBT. Numbers within the panel identify the values corresponding to the isopleths.

Figure 1 shows isopleths (lines of constant value) for two HSIs plotted on a chart of air temperature (*T*) and relative humidity (*h*). The same is shown using specific humidity in Supplementary Fig. 1. Although both UTCI and WBT are in units of °C, their values are not directly comparable. Firstly, they intersect at multiple points: for example, a WBT value of 20 °C could correspond to UTCI values ranging from just over 25 °C to over 40 °C for different combinations of *T* and *h* (Fig. 1). Secondly, they are clearly calibrated to different scales: the 35 °C isopleth in WBT approximately corresponds to the 55 °C isopleth in UTCI.

Visually comparing the gradients of the isopleths in Fig. 1 gives an intuitive way of comparing these indices. Where the line is closer to the vertical, the HSI is more sensitive to *T*; where the line is closer to the horizontal, the HSI is more sensitive to *h*. At high values (WBT = 35 °C and UTCI = 55 °C), the WBT and UTCI lines are nearly parallel: this means that the two indices agree on the relative importance of *T* and *h*, but only for these high values. For most of the space, the gradients of the lines are very different between the indices, showing that they disagree about the relative importance of marginal changes to *T* and *h*.

Making the gradient comparison numerically explicit enables easy identification of areas of disagreement. Consider a point (*T*, *h*); the HSI calculated at that point is *U*(*T*, *h*). Throughout this article, we use a scale of 0–100% for *h*. We will call the gradient of the isopleth of a heat stress function *U* at the point (*T*, *h*) the marginal temperature-equivalent change

(1) $$M=\frac{\partial U}{\partial T}/\frac{\partial U}{\partial h}$$

*M* can be interpreted as how much of a change in *h* produces a change in the HSI that is equivalent to a unit change in *T*. The smaller the value of *M*, the more sensitive the HSI is to changes in *h* compared to changes in *T*. In this study, the gradients are estimated using the forward difference approximation.

*M* can be compared freely between different HSIs under the same conditions (*T*, *h*) as the HSI is not one of the dimensions of *M*. More detail on the definition and calculation of *M* is given in the Methods section. This procedure is not restricted to the temperature–humidity space and could be equivalently performed for other variables, for example, in the temperature wind speed space.

**Fig. 2:**
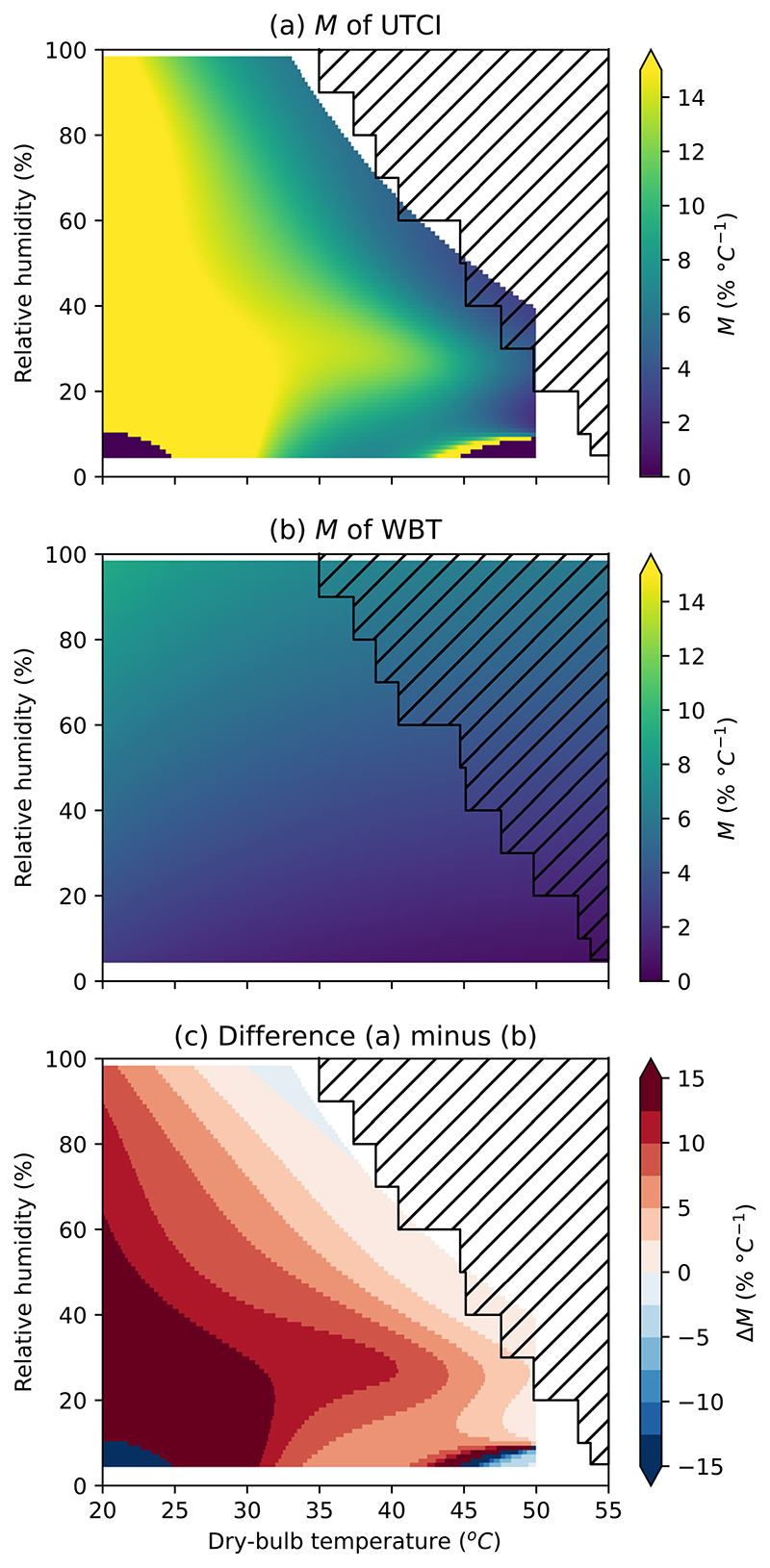
Marginal temperature equivalent changes (***M***) in relative humidity for a range of atmospheric conditions. For **a** UTCI and **b** wet-bulb temperature (WBT). Conditions in the hatched area did not occur from 1992 to 2022, according to ERA5. A high value of *M* (yellow) indicates that a large change in relative humidity is required to change an HSI by the same amount as a unit change in temperature. **c** *M* difference for UTCI minus WBT. A positive number (red) means that UTCI is less sensitive to relative humidity (as opposed to temperature) than WBT. A number close to zero (paler colours) indicates that the HSIs have similar responses. Supplementary Figure 2 shows the same but using specific humidity.

Figure 2 shows the *M* in *h* of UTCI and WBT. Supplementary Figure 2 shows the same in specific humidity. The hatched area shows atmospheric conditions that have not occurred in the period 1992–2022, according to ERA5. In Fig. 2a, the yellow shading to the left and bottom indicates where UTCI is most sensitive to changes in temperature. Conversely, the blue shading in the top right indicates where UTCI is more sensitive to humidity. For the yellow area, a change in temperature of 1 °C produces a change in the UTCI equivalent to a change in *h* of 15% or more. Contrast this with Fig. 2b: for WBT, *M* varies a little under different conditions.

To compare between different HSIs, we can take the difference in *M* between them. Figure 2c shows the difference in *M* (Δ*M*) between UTCI and WBT, subtracting Fig. 2b from Fig. 2a. In this case, a positive value of *ΔM* means that WBT is more sensitive to a change in *h*, while a negative value means that UTCI is more sensitive to a change in *h*. A value close to zero indicates that the HSIs have similar sensitivity to *h*. Generally, humidity is much more important for WBT than UTCI for low-temperature, low-humidity conditions, and the two HSIs agree under high-temperature, high-humidity conditions. UTCI and WBT are closest in *M* at around 35 °C WBT or 55 °C UTCI: *ΔM* is generally below 7.5 % for a WBT of >30 °C, below 5.0% for a WBT of 32 °C or above, and closest to zero around a WBT of 35 °C. In the present climate, WBT rarely exceeds 31 °C, but WBT > 35 °C occurs in some locations for a few hours at a time and could begin to occur at a large scale with global warming of around 7 °C^19,20^.

**Fig. 3:**
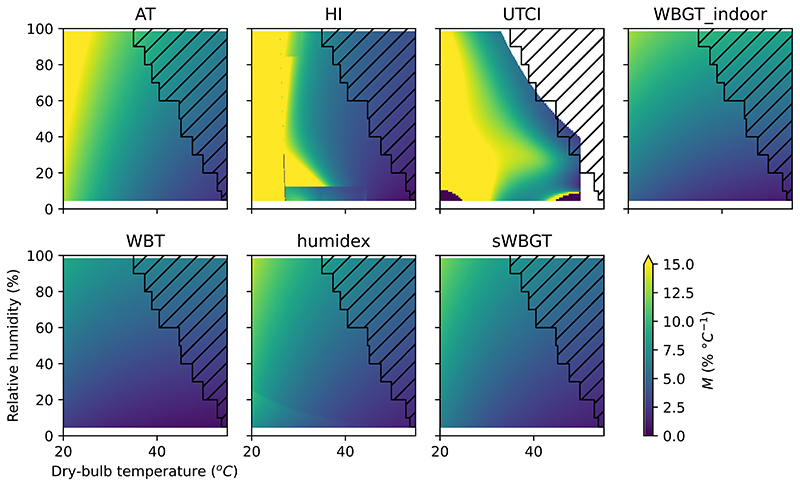
Marginal temperature equivalent changes (***M***) in relative humidity for a range of heat stress indices (HSIs) and atmospheric conditions. A high value of *M* (yellow) indicates that a large change in relative humidity is required to change an HSI by the same amount as a unit change in temperature. Conditions in the hatched area did not occur from 1992 to 2022, according to ERA5.

Figure 3 shows *M* for several commonly used HSIs. *M* differences between each of the variables are shown in Supplementary Fig. 4. We choose these HSIs to show a range of different outcomes, and this is not intended to be exhaustive. *M* enables comparisons between different conditions (e.g., between hot-humid and hot-dry conditions) as well as between different HSIs, even if calibrated to completely different scales. Patterns of agreement and disagreement between these HSIs are summarised by examining *M* in three regimes: the lower temperature regime, the hot-humid regime, and the hot-dry regime. These regimes are intended to be illustrative rather than definitive. The low-temperature regime is on the left-hand side of the plot, which we characterise as 20 °C temperature and under. The hot-humid regime is the top right-hand side of the plot, which we characterise at 35 °C temperature and 80% *h* (this is extremely hot and humid but occurs in the present-day climate). The hot-dry regime is on the bottom right-hand side of the plot, which we characterise as 40 °C with 20% *h*.

Figure 3 shows that AT, HI, and UTCI are mainly sensitive to temperature in the low-temperature regime. In fact, HI is defined as equal to temperature when below 26.7 °C (80 °F). In comparison, WBT has a very low *M*, even in the low-temperature regime. Humidex, WBGT-indoor, and sWBGT are slightly higher *M* in the low-temperature regime compared to WBT but clearly have lower *M* compared to AT, HI, and UTCI. Physiologically, we would not expect the effect of humidity to be important in the low-temperature regime: changes in the air temperature will dominate changes in heat balance as the sweat evaporation rate required to maintain heat balance is low^21^.

In the hot-dry regime of Fig. 3, we see a different pattern of disagreement between the HSIs. *M* is relatively low in AT, WBT, humidex, WBGT-indoor, and sWBGT. In contrast, HI and UTCI have a zone of relatively high *M*. In the hot-dry regime, the sweat evaporation rate required to maintain a stable body temperature is high, but sweat evaporation efficiency is also high as the vapour pressure is low^22^. Therefore, it makes sense physiologically that *M* is low. If sweat secretion is limited, then increasing the *h* (and therefore the vapour pressure) will not necessarily decrease the sweat evaporation rate as there is no more sweat to evaporate^22,23^. Sweat secretion rate limits in the underlying model explain the higher *M* values seen in the hot-dry regime for UTCI.

In the hot-humid regime, we would expect changes in *h* to be at their most important, as it is the regime where the vapour pressure is likely to be the limiting factor in sweat evaporation rate^22^. AT, HI, UTCI, WBT, humidex, WBGT-indoor, and sWBGT all have low *M* in the hot-humid regime, so they are roughly in agreement.

### Heat stress index selection can reverse the conclusion

In this section, we examine two illustrative examples of recent studies where the conclusions were strongly determined by the choice of HSI.

In Wouters et al.^11^, a combination of observations and atmospheric modelling is used to argue that soil droughts lead to a reduction in heatwave lethality. For selected heatwave events, they use atmospheric modelling to estimate what the effect would be if, at the start of the heatwave, the soil moisture had been at the mean climatological level for that location rather than its actual value. They find that higher soil moisture leads to lower temperatures and higher specific humidity. Based on Mora et al.^24^. they introduce a heat stress metric

(2) $$T_{s}=\text{ WBT }+4.5\left(1-h^{2}\right)$$

The counterfactual used means that some cases had soil moisture increased while others had soil moisture reduced; those which have soil moisture increased have increased specific humidity and vice versa. They find that lower soil moisture leads to lower values of *T_s_*, which they argue means soil drought reduces heatwave lethality. However, Table 1 from Wouters et al.^11^ we calculate changes in a variety of HSIs (Table 1) which highlights that some other HSIs have the opposite correlation with specific humidity, indicating that increased soil moisture would decrease heat stress. This does not necessarily mean that the choice of HSI in Wouters et al. is wrong, but it is clearly problematic that different HSIs give such opposing conclusions.

**Fig. 4:**
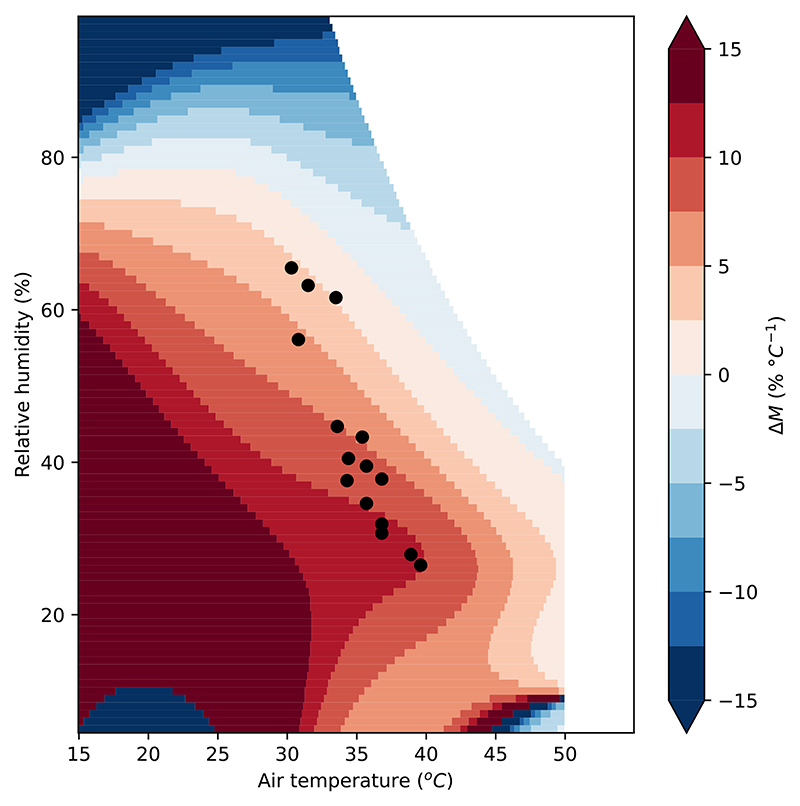
Heatwave atmospheric conditions from Wouters et al. (points). Difference in *M* of UTCI versus *T_s_* (shading): a positive number (red) means that UTCI is less sensitive to relative humidity (as opposed to temperature) than *T_s_* is. A number close to zero (paler colours) indicates that the HSIs agree. The points are all in the red shaded area, indicating that evaluating these changes using *T_s_* will place much more emphasis on changes in humidity than if they were evaluated with UTCI.

The reason for this can be understood by comparing *M* for different HSIs in the *T* and *h* range of interest for their study. Figure 4 shows the difference in *M* between *T_s_* and UTCI (shading), as well as the heatwave conditions studied by Wouters et al.^11^ (Table 1). In heatwave conditions, UTCI is much more sensitive to temperature (as opposed to relative humidity) compared to *T_s_*. It is possible to identify that there will be disagreement purely from the baseline atmospheric conditions without the need for any atmospheric modelling.

Another example is provided by Mishra et al.^12^, in which the effect of irrigation was modelled using the Weather Research and Forecasting regional climate model at 0.25° horizontal resolution driven by ERA5 reanalysis and covering the 2000–2018 period. Two scenarios were modelled: with and without irrigation. Mishra et al. found that irrigation decreases dry-bulb temperature but increases WBT, and therefore concluded that increased irrigation area can be detrimental to human heat stress. The accuracy of the assumptions Mishra et al. made about when and how much irrigation occurs have been discussed elsewhere^25^. The conclusion of Mishra et al. was in contrast with the interpretation provided by other studies, e.g., Thiery et al.^10^, which found that expansion of irrigation had a large enough effect of cancelling out the effect of global warming on temperature extremes in some regions. However, the results in Mishra et al. show that while 95th percentile WBT increased, 95th percentile temperature and HI decreased (Mishra et al. their Fig. 4). The increase in WBT was emphasised rather than the decrease in HI, which was not mentioned in the text.

We have highlighted two recent examples of papers which argued that an increase in soil moisture is generally harmful to heat stress due to an increase in humidity^11,12^. Taken at face value, these studies foreclose climate adaptations that would reduce temperature extremes while increasing humidity. But in both cases, using a different HSI would have reversed the conclusion, which demonstrates the importance of choosing the appropriate HSI. This is concerning from the perspective of climate change adaptation action: if we cannot identify what metrics are meaningful, then how are we to evaluate potential interventions?

In both Mishra et al.^12^ and Wouters et al.^11^, the choice of HSI was justified by regression of some measure of mortality against atmospheric conditions; however, in both cases, the solution chosen was not demonstrated to be better than other alternatives, and uncertainty was not considered. While the association between mortality and temperature is well established by epidemiological studies, research into the effect of humidity on mortality has yielded conflicting results: variously finding a protective effect from humidity^26^, a U-shaped relationship between humidity and mortality^27^, or that no HSI is consistently better than others^28^. This may be resolved in future as more multi-country epidemiological studies are performed.

### Applicable conditions of HSIs

Certain HSIs aim to be rationally based on more detailed assumptions about the human body (especially UTCI, HI, and humidex). However, the complexity of these HSIs means it may be difficult to understand the differences between them. Our *M* method allows the properties of HSIs to be compared in terms of their output rather than the process used to derive the HSI.

The creation of any HSI requires simplifying assumptions which limit general applicability. These might include assumptions about the human body, physical activity, or meteorological conditions. UTCI and HI are both designed to represent a person of a certain fixed height and weight, walking at a fixed speed, and so may not represent clinically vulnerable or occupationally vulnerable populations well. Many HSIs have limited ranges in temperature and humidity outside of which they are undefined, which can be problematic when applied globally or to climate extremes: UTCI is undefined for vapour pressure above 5 kPa or air temperature above 50 °C^29^, and NOAA’s formula for the HI is poorly extrapolated at high temperature and humidity^30^. These limitations are not always widely known or correctly applied. Conditions for which UTCI is undefined already occur in the present climate (1992–2022) in ERA5.

On the other hand, by using WBT as a heat stress index, one represents a person as a well-ventilated wet surface. The use of WBT has been defended through its status as a thermodynamic quantity and through the fact that it does not make assumptions that are specific to a subset of the human population. However, the result is that it only represents human thermophysiology to a very extreme limit. This has especially problematic implications for studies involving soil moisture: by definition, adiabatic evaporation occurs at constant WBT^31^, so it is impossible to change WBT directly by adding moisture directly to an air parcel. Therefore, any study into the effects of vegetation or soil moisture changes is likely to observe WBT staying the same or increasing with soil moisture, regardless of whether the environment is wet or dry. This will lead to the inappropriate conclusion that soil moisture always increases heat stress.

### Recommendations

Given that all HSIs are designed for a specific use and context, heat indices would ideally be tailored to a specific impact and population under study^32^, but this is not always practical. HSIs do not have to be based on physiological assumptions if there is strong empirical justification for their use. For example, a study to find the most appropriate HSIs for use in occupational settings identified WBGT and the UTCI as the best, based on 17 criteria derived from an expert consultation process (e.g., the correlation coefficient between the HSI and body core temperature) evaluated against field data from multiple countries and occupational environments^33^.

However, if physiological justifications are used for a particular HSI, then the HSI used must correctly reflect physiology. For example, atmospheric chamber studies have shown that heat stress response to WBT is not consistent between high humidity and low humidity environments, making its global application problematic^34,35^. It may be preferable to use UTCI with simplified assumptions (that wind is fixed and mean radiative temperature has a fixed relationship to air temperature) rather than using WBT, as this better reflects physiology. But UTCI has its own limitations: it is derived from a modelled 74 kg person walking at 1.1 ms^−1^, which cannot represent all people and activities, and is undefined for very high temperatures and humidities. More flexible models of human heat balance offer another solution^30,36^, but are more complex computationally and conceptually.

Our *M* method provides a straightforward and rational basis for comparisons between HSIs. Researchers can identify the range of temperature and relative humidity most relevant to their study, then examine Fig. 3 to identify whether there is likely to be disagreement between different HSIs in that range. We expect that in many cases, it will be sufficient to compare results using HI, UTCI, and WBT as these cover most of the variation shown in Fig. 3. Calculating and reporting multiple HSIs can be useful, especially if disagreement is identified as a result.

In all cases, it is best practice to report component measurements as well as composite indices. For example, if reporting the 95th percentile HI, one should also report the temperature and humidity corresponding to that value (which will not necessarily be the 95th percentile of temperature and humidity separately), as this allows the reader to make conversions to other HSIs. If a change in WBT is reported, the baseline value of WBT, as well as the corresponding baseline and change values of dry-bulb temperature, need to be reported for it to be meaningful.

In this study, we presented a more accessible numerical comparison of HSIs than previous studies and encouraged researchers to critically assess how their findings may be dependent upon their choice of HSIs. Furthermore, we presented examples where the choice of heat stress metric reversed the conclusions of studies on the effect of soil moisture on heat stress. This is especially important in cases where a joint change in temperature and humidity is considered, for example, expanding irrigation or urban greening. We argue that the choice of HSI needs to be specifically justified by the domain of study and that it must be reported and addressed where conclusions are reversed by choice of HSI. We also encourage researchers to report component measurements (temperature and humidity) alongside their HSIs, as this allows conversion between different HSIs.

## Methods

### Calculation of heat stress indicators

WBT measures the coldest temperature that can be achieved by evaporative cooling at a given temperature and humidity. WBT was calculated using the Davies–Jones method^37^.

Apparent temperature, HI, and humidex are based on heat balance principles that incorporate temperature and humidity; care must be taken as multiple specifications exist^3^. HI was calculated using the NOAA specification from https://www.wpc.ncep.noaa.gov/html/heatindex_equation.shtml. The apparent temperature was calculated based on Steadman’s shaded specification^16^, which we found in Ioannou et al.^3^. Humidex is defined by the Meteorological Service of Canada; we used the equation listed in Ioannou et al. Some specifications of AT incorporate wind speed and radiation; we used one incorporating wind (assumed to be 0.5 ms^−1^) but not radiation.

UTCI is based on detailed modelling of heat balance^38^, incorporating temperature, humidity, wind speed and radiation; it is less commonly used in heat-health studies, which may be due to the requirement for radiant temperature input. UTCI was calculated using the UTCI polynomial, which is described in Bröde et al. and available on the web from www.utci.org. Wind Speed at 10 m was assumed to be 0.5 ms^−1^, which is the minimum value for which reference conditions were included^29^. The limits specified in Brode et al. were applied to the UTCI polynomial^29^, a step which is sometimes forgotten.

WBGT was developed as a field measurement for use by the military in hot-humid environments and incorporated temperature, humidity (via WBT) and radiation (via black globe temperature)^39^. Simplified WBGT is sometimes used in heat-health studies and is intended as an approximation of WBGT at the moderately high wind and moderately high radiation levels; WBGT is often calculated without the radiation component leading to a quantity referred to as WBGT-indoor^17^. WBGT-indoor was calculated in line with Eq. 2 in Lemke and Kjellstrom^39^, where WBT was again calculated using the Davies-Jones method. sWBGT was calculated using the equation listed in Ioannou et al. Compared to more detailed calculations of WBGT, sWBGT is a poor approximation but is still frequently used^40^.

When the mean radiative temperature was required, it was assumed to be equal to the air temperature; this is thought to be reasonably accurate for indoor conditions^21^ but not for outdoor conditions. In Supplementary Fig. 4, we show that if, instead, the mean radiative temperature is assumed to be higher than air temperature by a constant increment or if the wind speed is assumed to be higher, then the *M* pattern of UTCI remains similar, and *M* generally increases.

### Calculus of variations

Consider a point in the temperature-humidity space (*T*, *h*) where *T* is temperature and *h* is humidity; the HSI calculated at that point is *U*(*T*, *h*). Increasing the temperature by a small amount *δT*, will change the heat stress to *U*(*T* + *δT*, *h*) assuming humidity remains the same. Similarly, increasing the humidity by a small amount *δh* will change the heat stress to *U*(*T*, *h* + *δh*).

If *δT* were fixed and *δh* were solved to give the same change in the HSI *U*, i.e., solving

**Fig. 5:**
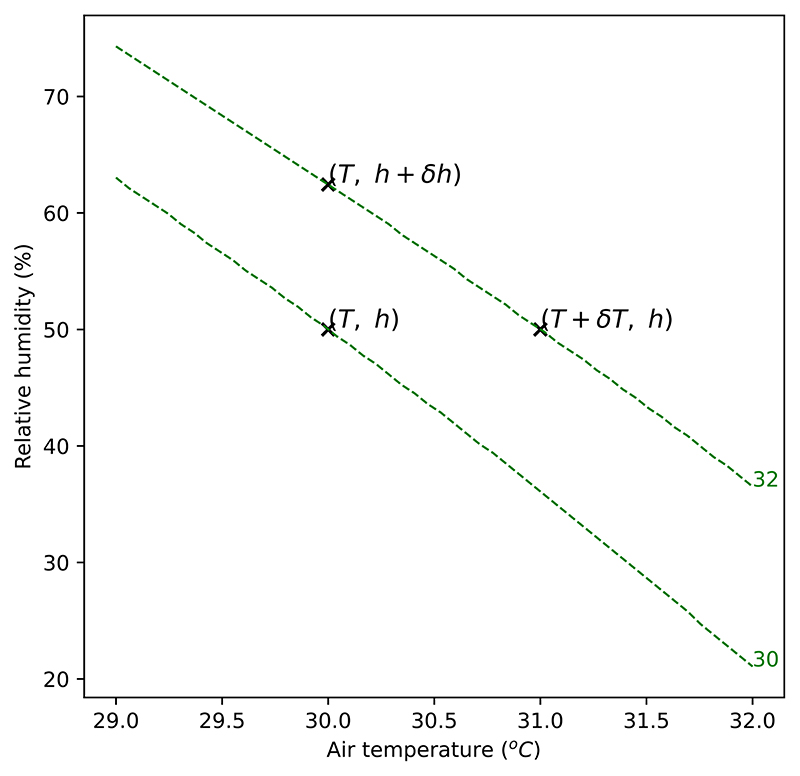
Points in the space of temperature and humidity. Lines are isopleths of a heat stress index. The marginal temperature equivalent change is the change in humidity which produces a change in the HSI equivalent to a unit change in temperature.

(3) U(T+δT,h)−U(T,h)=U(T,h+δh)−U(T,h)

for *δh* at fixed *δT*, then the quantity $\frac{\delta h}{\delta T}$ would tell us what change in humidity would produce a change in *U* equivalent to a 1 °C change in temperature. This is illustrated in Fig. 5. If *δT* and *δh* are small, then this is equivalent to the ratio of partial differentials

(4) $$\frac{\delta h}{\delta T}=\frac{\partial U}{\partial T}/\frac{\partial U}{\partial h}$$

It is more practical numerically to estimate $\frac{\partial U}{\partial T}$ and $\frac{\partial U}{\partial h}$ directly using a finite differences method, rather than solving the equation. If *δT* and *δh* are small, then we can estimate the one-dimensional gradients of the function *U*(*T*, *h*) using the forward difference approximation:

(5) $$\left(U(T,h)-U(T+\delta T,h))/\delta T\approx \frac{\partial U}{\partial T}(T,h\right)$$

and

(6) $$\left(U(T,h)-U(T,h+\delta h))/\delta h\approx \frac{\partial U}{\partial h}(T,h\right)$$

We choose to do this numerically because not all the HSIs are analytically differentiable, and we choose this approximation because it is easiest to explain.

Generally, we will refer to *h* in this paper as it will be more familiar to most readers, although the Clausius–Clapeyron relation complicates its comparison at different temperatures; key figures are repeated using specific humidity in the Supplementary Material, and the results using either is consistent. Calculations are made with all else being equal, so there is implicitly a small change in the specific humidity of the air parcel if *h* is held constant with changing temperature and vice versa. The marginal temperature-equivalent change procedure can be applied whether humidity is represented as *h* or any other measure of humidity.

In this study, we have only compared the effect of temperature and humidity for a non-exhaustive selection of commonly used HSIs. Radiation and wind are important components of heat stress but are beyond the scope of this paper. The effect of different calculation assumptions on the estimated effect of radiation could be an important avenue for future investigation which is also amenable to the *M* approach.

### Limits of present climate

The limits of the present climate were estimated from ERA5 reanalysis^41^. Hourly reanalysis data from 1992–2022 were used. First, relative humidity was calculated from 2 m air temperature and 2 m dewpoint temperature. Then, for each relative humidity in the maximum co-occurring 2 m temperature was found.

## Supplementary Material

Supplementary information The online version contains supplementary material available at https://doi.org/10.1038/s41612-023-00408-0.

SUPP 1

## Figures and Tables

**Table 1:** Correlation between specific humidity changes and heat stress index changes.

	Kendall	Spearman
*T_s_*	0.66	0.83
WBT	0.85	0.94
UTCI	−0.85	−0.95
WBGT-indoor	0.18	0.24
Humidex	0.33	0.50
Heat index	−0.30	−0.37
sWBGT	0.64	0.75
Apparent temperature	−0.50	−0.61

Rank correlation (Kendall’s tau and Spearman’s *R*) between the changes in specific humidity and the changes in heat stress indices (HSIs) calculated from the temperature and humidity changes induced by soil moisture changes modelled by Wouters et al. A positive number means that modelled changes to soil moisture that increased humidity and decreased temperature led to an increase in that HSI. While the changes in the HSI used in Wouters et al. are positively correlated with the corresponding changes in humidity, other commonly used heat stress indices have weaker or opposite correlations.

## Data Availability

Data are freely available for download. The estimated limits of the ERA5 reanalysis are included in the archive available at https://doi.org/10.5281/zenodo.8038787. No other data were generated by this study.

## Code Availability

The full Python code used in this analysis is archived and freely available for download from https://doi.org/10.5281/zenodo.8038787.
