# Supplementary material for: Commonly used indices disagree about the effect of moisture on heat stress
Source: NPJ Clim Atmos Sci. Author manuscript; Available in PMC 2024 Jan 10. (PMC7615504; doi:10.1038/s41612-023-00408-0)
Supplement: SUPP 1 [file EMS192600-supplement-SUPP_1.pdf]

Commonly used indices disagree about the effect of moisture on heat stress

Charles Simpson*[1] (corresponding author), Oscar Brousse[1], Kristie Ebi[2], Clare Heaviside[1],

1 Institute of Environmental Design and Engineering, Bartlett School of Environment Energy and Resources, University College London, 14 Upper Woburn Place, London, United Kingdom

2 Center for Health and the Global Environment, University of Washington, Seattle WA USA

* Corresponding author email: charles.simpson@ucl.ac.uk

Supplementary Material

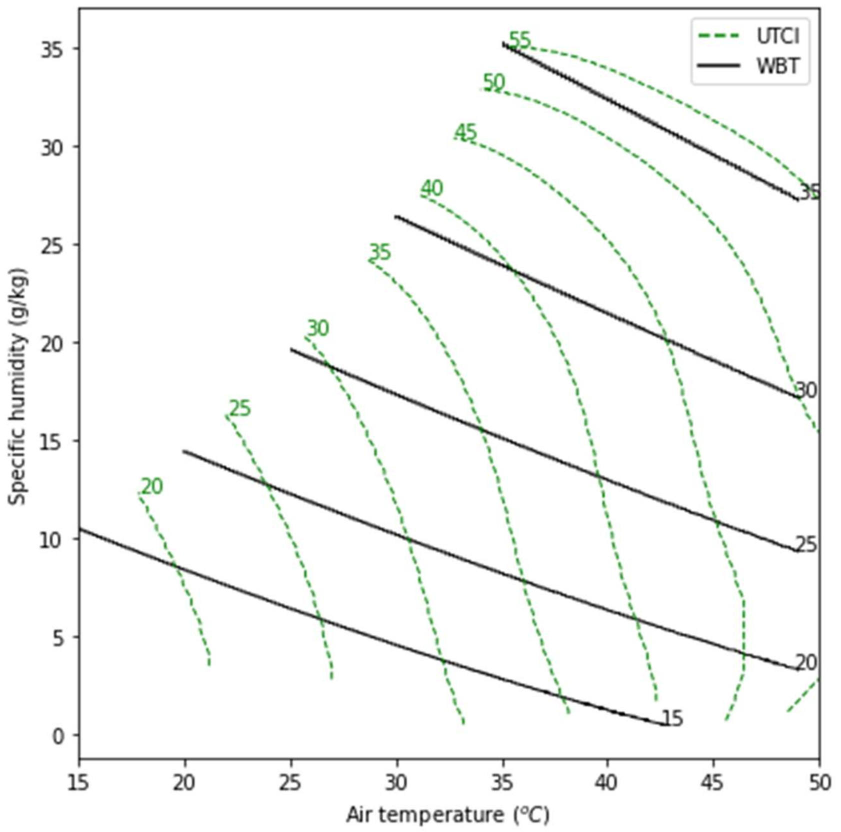

Supplementary Figure 1: As Figure 1, but using specific humidity instead of relative humidity. Isopleths of UTCI (dashed green) and WBT (solid black) in temperature-humidity space.

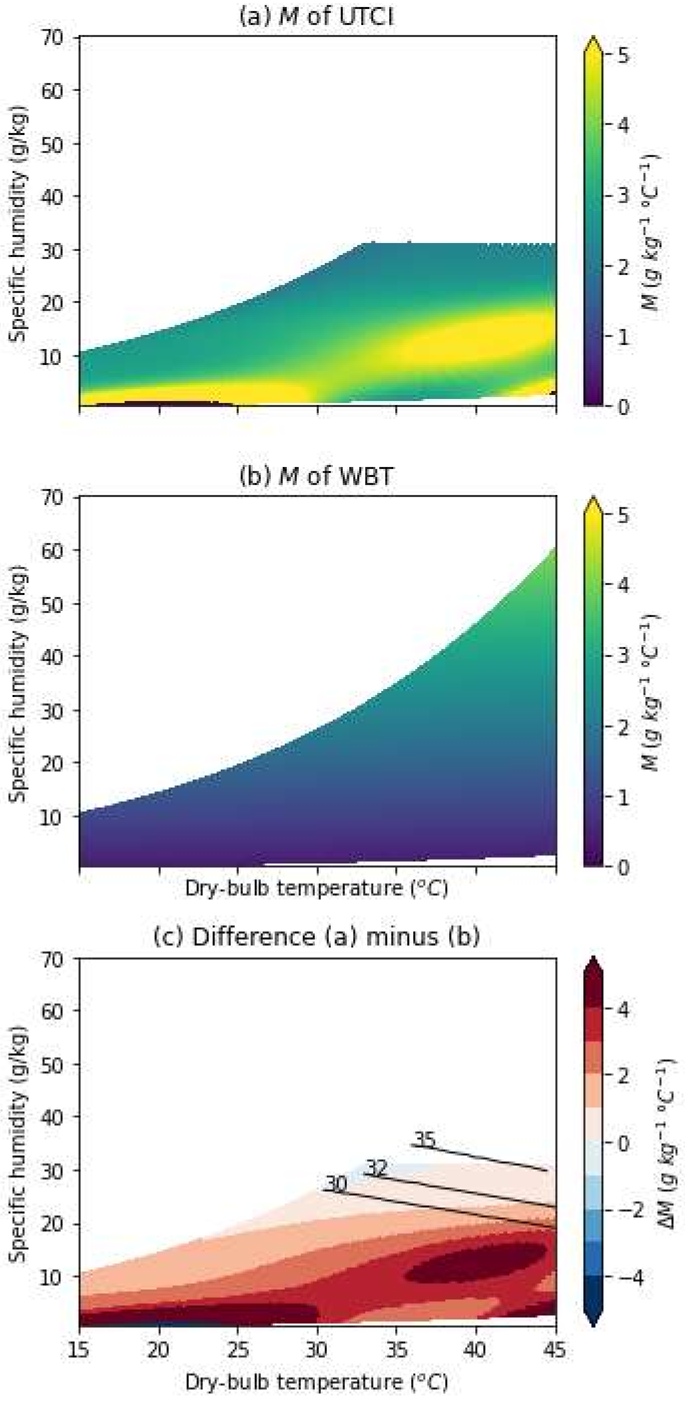

Supplementary Figure 2: As Figure 2, but using specific humidity instead of relative humidity. Marginal temperature equivalent changes (*M*) in specific humidity for a range of atmospheric conditions for (a) UTCI and (b) wet-bulb temperature (WBT). A high value of *M* (yellow) indicates that a large change in specific humidity is required to change a HSI by the same amount as a unit change in temperature. (c) shows *M* difference for UTCI minus WBT. Black solid lines are isopleths of WBT. A positive number (red) means that UTCI is less sensitive to specific humidity (as opposed to temperature) than WBT is. A number close to zero (paler colours) indicates that the HSIs agree.

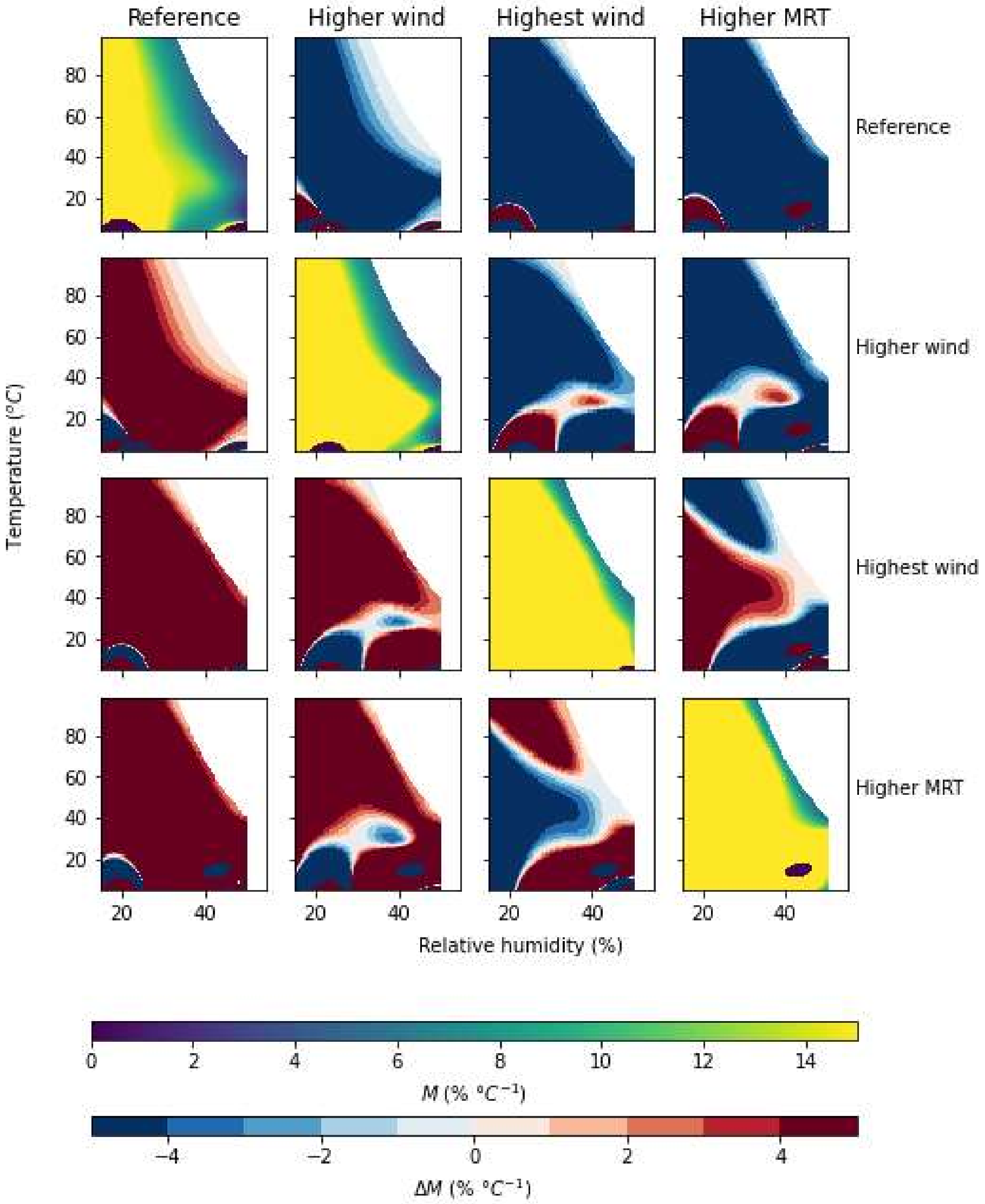

Supplementary Figure 3: Marginal temperature equivalent change (*M)* difference plots between all UTCI under different simplifying assumptions. Plots on the diagonal show absolute values of *M*, whereas off diagonal plots show the difference in *M* (row minus column). *Reference* refers to the assumption that windspeed = 0.5 m/s, mean radiant temperature (MRT) = air temperature (Ta). *Higher wind* assumes that windspeed is instead 5 m/s. *Highest wind* assumes instead that windspeed is 30 m/s. *Higher MRT* assumes that MRT = Ta + 30 °C. Increased windspeed or MRT increases *M*, i.e. makes UTCI less sensitive to humidity.

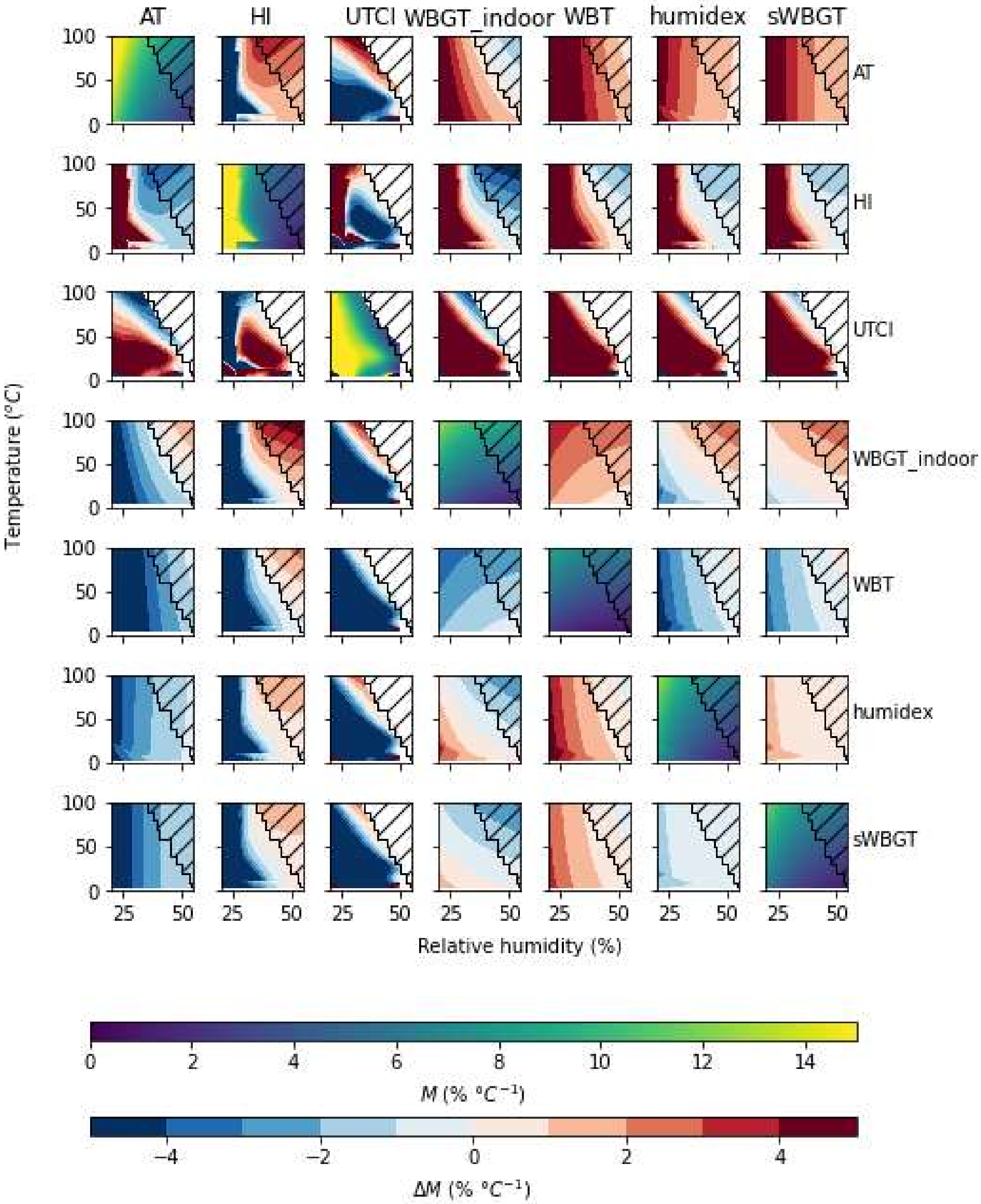

Supplementary Figure 4: Marginal temperature equivalent change (*M*). difference plots between all HSIs discussed in the text. Plots on the diagonal show absolute values of *M*, whereas off diagonal plots show the difference in *M* (row minus column). Conditions in the hatched area did not occur 1992-2022 according to ERA5.
